# Evaluation of* LATS1* and* LATS2* Promoter Methylation with the Risk of Pterygium Formation

**DOI:** 10.1155/2016/5431021

**Published:** 2016-01-28

**Authors:** Maryam Najafi, Dor Mohammad Kordi-Tamandani, Mohammad Arish

**Affiliations:** ^1^Departement of Biology, University of Sistan and Baluchestan, Zahedan, Iran; ^2^Department of Ophthalmology, Al-Zahra Eye Hospital, Zahedan University of Medical Sciences, Zahedan 98167-43463, Iran

## Abstract

*Purpose*. Pterygium is a serious eye problem in countries with high exposure to UV. However, despite numerous studies, the molecular etiology of pterygium is unclear. Recent studies have indicated that* LATS1* and* LATS2* genes are involved in DDR signaling pathways against continuous UV exposure. Our aim was to evaluate the* LATS1* and* LATS2* promoter methylation with the risk of pterygium formation.* Methods*. We evaluated the promoter methylation status of* LATS1* and* LATS2* using methylation-specific PCR technique. Also, mRNA expression of* LATS1* and* LATS2* was assessed in 14 cases of pterygium and 14 normal specimens by real-time PCR.* Results*. Promoter methylation of* LATS1* and* LATS2* was detected significantly between pterygium tissues and normal tissues [*LATS1*; OR = 4.9; 95% CI: 1.54 to 15.48, *P* = 0.003;* LATS2*; OR = 7.1; 95% CI: 1.53 to 33.19, *P* = 0.004]. The gene expression analysis showed a statistically significant difference between pterygium tissues and healthy controls for both* LATS1* and* LATS2* (*P* < 0.05).* Conclusions*. The data of this study is the first report regarding the effect of promoter methylation of the* LATS1* and* LATS2* in the pterygium. To confirm these data, doing further studies in various genetic populations with large sample sizes using advanced molecular techniques is proposed.

## 1. Introduction

**Figure 1:**
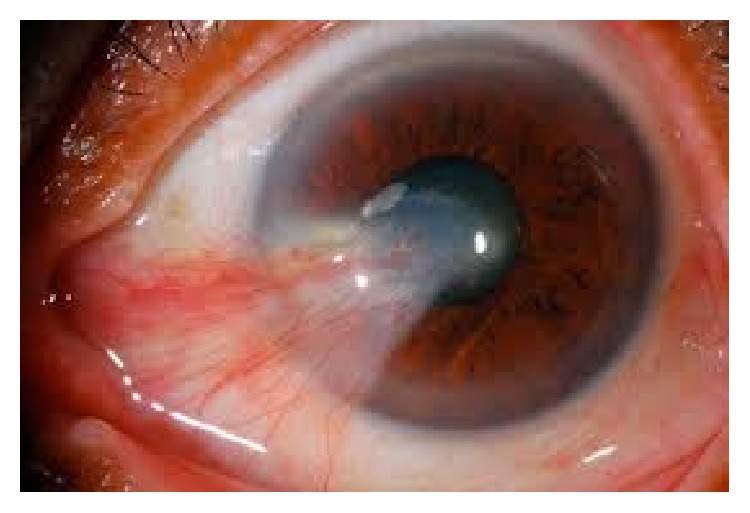
Pterygium.

Pterygium is a common wing-shaped and oriented fibrovascular lesion coating the surface of the eye. According to the population-based studies, its prevalence rate varies from 0.7% to 33% [1]. This abnormality arises from the conjunctiva and extends into the cornea and can result in remarkable cosmetic problems, visual impairment, recurrent inflammation, and mild irritation [2]. Surgery is needed for cases when lesion expands to the central part of the cornea [3] (Figure 1).

There are countless theories regarding the causes of pterygium including UV light exposure, viruses, oxidative stress, DNA methylation, apoptotic and oncogenic proteins, loss of heterozygosity, microsatellite instability, inflammatory mediators, extracellular matrix modulators, lymphangiogenesis, cell epithelial-mesenchymal transition, and alterations in cholesterol metabolism [4]. Most studies have implicated that pterygium is a UV light associated disease. Therefore, we focused on* LATS1* and* LATS2* genes which are the common tumor suppressor genes in the UV-induced DNA Damage Response (DDR) signaling pathways.* Lats* (large tumor suppressor) gene, a Ser/Thr kinase, belongs to the Ndr/LATS subfamily of AGC (protein kinase A/PKG/PKC) kinases originally isolated from* Drosophila melanogaster*. Two mammalian homologs of fly* lats*,* LATS1* and* LATS2*, are located in 6q25.1 and 13q12.11 chromosomes, respectively [5].

One of the DDR signaling pathways, which facilitate apoptosis following high levels of UV-induced damage, is* Chk1-Lats2-p21* axis [6]. And* Chk1-Lats2-(14-3-3)* regulates the P-body formation as a unique signaling pathway in response to UV-induced DNA damage [7].* LATS1* and* LATS2* are also engaged in the regulation of cell cycle through G2-M arrest and G1-S arrest, respectively [8, 9]. After DNA damage the integrity of genome is warranted through* RASSF1A-LATS1/2-MDM2-P53* signaling pathway [10]. In addition, a large amount of literature has reported the function of these genes in morphogenesis, cell division, and apoptosis [11].

Epigenetic modifications such as DNA methylation of CpG islands in promoter regions are the main cause of tumor suppressor gene silencing and can result in tumor development [12]. Some tumors such as breast cancer and astrocytoma have shown downregulation of* LATS1* and* LATS2* mRNA expression through promoter methylation [13]. To our knowledge for the first time, this study highlights the status of* LATS1* and* LATS2* promoter methylation and mRNA expression profiles in pterygium.

## 2. Materials and Methods

### 2.1. Subject

This case-control study was performed from 2010 to 2013 consisting of 70 primary pterygium tissues (35 males and 35 females with a mean age of 52.44 ± 20.611) and 70 normal conjunctiva tissues of the patients who had undergone cataract surgery (35 males and 35 females with a mean age of 50.67 ± 23.318). The biopsy tissue samples were frozen in −80°C until molecular analysis. These samples were collected from Al-Zahra Eye Hospital. All procedures in this study were approved by the Ethical Board at the Zahedan University of Medical Sciences. Informed consent was taken from all participants. Arish et al. 2016 described the clinical information of the patients who have participated in this study [14].

### 2.2. DNA Extraction and Modification

Genomic DNA was extracted from frozen tissues by phenol chloroform isoamyl alcohol extraction protocol. Then 1-2 *μ*g of isolated DNA was diluted in 20 *μ*L of water and used for bisulfite treatment by Wizard DNA Clean-Up System (Promega) kit which converts unmethylated cytosine to uracil and leaves methylated cytosine unaltered. According to the manufacturer's instructions of Promega, the treated DNA should be diluted in 20 *μ*L of water and kept at −20°C for using in the further experiments.

### 2.3. Methylation-Specific PCR (MSP)

**Figure 2:**
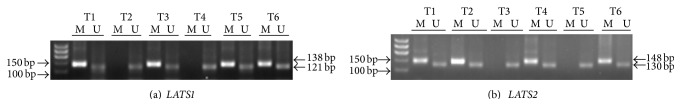
Representative results of methylation-specific PCR analysis of* LATS1* (a) and* LATS2* (b) in random pterygium tissues (T). M, methylated; U, unmethylated. Marker, 50 bp size marker.

To carry out the MSP analysis, promoters of the genes were recognized through online data analysis (http://www.ensembl.org) and then the preferred sequences were used to design methylated and unmethylated primers by MethPrime online software. Our selection for the site of methylated and unmethylated was consistent with the related literature.* AccuPower* HotStart PCR Premix from Bioneer Company (Cat. Number: K-5050) was used for each PCR reaction. Each PCR reaction contained 1 *μ*L of modified DNA and 0.5 *μ*L of each primer which is dissolved in the lyophilized blue pellet of* AccuPower* HotStart PCR Premix reached a final volume of 20 *μ*L with water. The MSP amplification was set as follows: 95°C for 5 min, followed by 40 cycles (95°C for 40 s, the annealing temperature for* LATS1*: M = 53, U = 57;* LATS2*: M = 55.5, U = 55 for 40 s and extension at 72°C for 40 s). Final incubation was completed at 72°C for 10 min. The designed primers were listed in Table 1. PCR products were detected by electrophoresis in 2% agarose gel, 80–100 volts for an hour until being well separated (Figure 2).

#### 2.3.1. RNA Extraction and Modification

Total RNA was extracted from pterygium and control tissues using the RNX-Plus solution (Cat. Number: MR7713C). A Revert Aid First Strand cDNA Synthesis Kit (Fermentas, Cat. Number: K1621) was used to reverse-transcribe 1 mg of RNA to cDNA in a final volume of 20 *μ*L.

#### 2.3.2. mRNA Quantification by Real-Time PCR

Real-time PCR was performed using SYBR green in ABI 5700 sequence detection system (Applied Biosystems). We compared the mRNA expression in pterygium tissues related to normal tissues. 18S-rRNA was used as an internal standard. PCR efficiencies (*E*) were calculated for all used primers from the given slopes of standard curves, generated from serial dilutions of positive controls, according to the following equation: *E* = 2^(−ΔΔCT)^ [15]. The designed primers for expression analysis are shown in Table 2.

### 2.4. Statistical Analysis

The effect of* LATS1* and* LATS2* genes methylation on the risk of pterygium formation was detected by estimating odds ratios (OR) and 95% confidence intervals (95% CI), using Logistic Regression. Avoiding bias in estimating OR, we calculated confidence intervals by three methods including exact, Cornfield, and Woolf. The Stata SE (version 13.1) was employed for statistical analyses. The Mann-Whitney test was used to compare expression data between groups. The significance level was set at *P* ≤ 0.05.

## 3. Results

The methylation frequency of* LATS1* gene was 66 (94.28%) for cases and 54 (77.14%) for healthy controls.* LATS2* gene showed 98.57% (N = 69) methylation in cases and 82.86% (N = 58) in the controls group. Comparison of methylated versus unmethylated indicated significant difference between cases and controls in* LAST1* (OR = 4.9; 95%CI: 1.54 to 15.48, *P* = 0.003) and* LATS2* (OR = 7.1; 95%CI: 1.53 to 33.19, *P* = 0.004) (Table 3).

Decreased expression in case group of both candidate genes (0.42 ± 0.030 in case versus 0.57 ± 0.068 for controls in* LATS1* and 0.44 ± 0.028 in cases versus 0.57 ± 0.061 controls in* LATS2*) was detected. Comparison of mean between cases and controls revealed a statistically significant difference in both genes (*P* < 0.05) (Table 4).

## 4. Discussion

Pterygium is a benign lesion that extends from conjunctiva to the cornea where it may interfere with vision. Since the current treatment of pterygium is invasive, mainly based on surgery, studies for new markers should be conducted. The current knowledge regarding the molecular basis of pterygium needs to be widened. Presently pterygium is considered as a UV light exposure-related uncontrolled cell proliferation [16]. Environmental factors such as UV radiation play an important role in tumor promotion through the epigenetic dysregulation of the cell cycle genes [17].

*LATS1* and* LATS2* as a part of DDR signaling pathways are putative serine/threonine kinase proteins that localize to the mitotic apparatus and constitute a complex with cell cycle control system [9, 18].* LATS1* acts as a negative regulator of* CDC2/cyclinA*, which reduces H1 histone kinase activity of* CDC2* and results in a G2-Mcell-cycle arrest [19]. Also,* LATS1* is activated by* RASSF1A* (*Ras* association domain family 1 isoform A) that stimulates response to DNA damage [20]. The activation of* LATS1* promotes genomic stability via stabilizing replication forks by restricting* CDK2*-mediated phosphorylation of* BRCA2*. This modulation not only has a fundamental role in error-free DNA repair but also maintains nucleofilament formation at stalled replication forks [21].* LATS2*  localizes to centrosomes during interphase, both early and late metaphase. It interacts with the centrosomal proteins aurora-*A* and ajuba and also is required for the accumulation of gamma-tubulin and spindle formation at the onset of mitosis [18]. It also interacts with a negative regulator of* p53* and may function in a positive feedback loop with* p53* that responds to the cytoskeleton damage. The* Lats2-Mdm2-p53* axis thus constitutes an innovative checkpoint pathway critical for the maintenance of proper chromosome number [22]. Both* LATS1* and* LATS2* as tumor suppressors are part of hippo signaling pathway which has profound effects on normal cell fate and tumorigenesis [23]. Therefore, silencing of* LATS1* and* LATS2* putative tumor suppressor genes through promoter methylation may cause the development of pterygium. The methylation of* LATS1/LATS2* has been demonstrated in Japanese lung cancer patients [24]. Decreased expression of* LATS1* in colorectal cancer was in association with the promoter methylation [25]. In addition, promoter hypermethylation mediates decreased expression of* LATS1* and* LATS2* in human astrocytoma [13]. Downregulation of* LATS1* and* LATS2* mRNA expression by promoter hypermethylation has been reported in breast cancer [5]. Consistent with the abovementioned studies, our results confirmed the significant relationship between reduced expression of the* LATS1* and* LATS2* through methylation and the risk of pterygium formation. Besides our data, the literature reviews showed the aberrant DNA methylation and decreased expression of* P16*,* Ecadherin*,* TGM 2, MMP2*, and* CD24* genes in pterygium [26–28]. Exploring pterygium methylation markers and their subsequent effects on mRNA expression paves the road for better therapy such as discovering drugs with the regulation of methylation characteristic. Further studies are required to identify the exact molecular function of* LATS1/LATS2* genes in pterygium in various and larger genetic populations using advanced molecular techniques.

## Figures and Tables

**Table 1:** Methylation primer sequences and annealing temperature.

Genes	Sequences (5′-3′)	Annealing temperature (°C)	Product size
*LATS1* M	F: GGAGTTT CGTTTTGTC	53°C	138 bp
	R: CGACGTAATAACG AACGCCTA		

*LATS1* U	F: TAGGTTGGAGTGTGGTGGT	57°C	121 bp
	R: CCC AACATAATAACAAACACCT		

*LATS2* M	F: ATTTCGGTTTATTGTAATTTTC	55°C	148 bp
	R: AACCAACATAATAAAACCCCG		

*LATS2* U	F: TTTGTTTTTTGGGTTTAAGT	55°C	130 bp
	R: CCAACATAATA AAACCCCA		

M, methylated; U, unmethylated.

**Table 2:** Expression primer sequences and annealing temperatures.

*LATS1*	F: GTTAAGGGGAGAGCCAGGTCCTT	60°C	132 bp
	R: TCAAGGAAGTCCCCAGGACTGT		

*LATS2*	F: ACTTTTCCTGCCACGACTTATTC	60°C	77 bp
	R: GATGGCTGTTTTAACCCCTCA		

*18SRNA*	F: GTAACCCGTTGAACCCCATT	60°C	112 bp
	R: CCATCCAATCGGTAGTAGCG		

**Table 3:** Risk of pterygium formation based on gene promoter methylation^a^.

Gene	Methylation status	Pterygium tissues *n* = 70	Normal tissues *n* = 70	OR	95% CI	*P*
		*n* (%)	*n* (%)			
Exact method						
*LATS1*	U (ref)	4 (5.71)	16 (22.86)	4.9	1.44 to 21.06	0.003
	M	66 (94.28)	54 (77.14)			
*LATS2*	U (ref)	2 (2.85)	12 (17.14)	7.1	1.47 to 67.42	0.004
	M	69 (98/57)	58 (82.86)			

Cornfield method						
*LATS1*	U (ref)	4 (5.71)	16 (22.86)	4.9	1.60 to 14.76	0.003
	M	66 (94.28)	54 (77.14)			
*LATS2*	U (ref)	2 (2.85)	12 (17.14)	7.1	1.70 to NC	0.004
	M	69 (98/57)	58 (82.86)			

Woolf method						
*LATS1*	U (ref)	4 (5.71)	16 (22.86)	4.9	1.54 to 15.48	0.003
	M	66 (94.28)	54 (77.14)			
*LATS2*	U (ref)	2 (2.85)	12 (17.14)	7.1	1.53 to 33.19	0.004
	M	69 (98/57)	58 (82.86)			

^a^Binary logistic regression analysis.

U: unmethyl, ref: reference, and M: methyl.

OR = odds ratio; 95% CI = 95% confidence interval.

NC: not calculated.

**Table 4:** Comparison of relative gene expression for *LATS1* and *LATS2* genes between patients with pterygium and healthy controls.

Genes		Number	Mean ± SD	*P* value^a^
*LATS1*	Cases	14	0.42 ± 0.030	<0.001
	Controls	14	0.57 ± 0.068	

*LATS2*	Cases	14	0.44 ± 0.028	<0.001
	Controls	14	0.57 ± 0.061	

^a^Mann-Whitney *U* test.
